# A case of abdominal textiloma following gynecologic surgery at the Yaounde Central Hospital, Cameroon

**DOI:** 10.11604/pamj.2013.16.147.3201

**Published:** 2013-12-20

**Authors:** Florent Ymele Fouelifack, Jovanny Tsuala Fouogue, Jeanne Hortence Fouedjio, Zacharie Sando

**Affiliations:** 1 Department of Obstetrics and Gynecology of the Yaoundé Central Hospital – Cameroon; 2 Research, Education and Health Development Associate's Group (REHDAG), Dschang, Cameroon; 3 Faculty of Medicine and biomedical Sciences of the University of Yaoundé I – Cameroon; 4 Head of the Unit of the Yaoundé Gyneco-obstetrics and Pediatric Hospital, Cameroon

**Keywords:** Textiloma, surgery, Cameroon

## Abstract

Textiloma is the inadvertent retention of a textile foreign body on the surgical site. It is a rare complication of surgery but which carries severe consequences for both patients and surgeons in terms of morbi-mortality and medico-legal procedures respectively. We herein report the case of an abdominal textiloma in a 42 year old woman who underwent a total abdominal hysterectomy for symptomatic leiomyomas. We also depict the errors that led to this mishap in a tertiary hospital in Yaounde (Cameroon). The textiloma was recognized six weeks after the causative surgery and removed by laparotomy without further complications.

## Introduction

Retention of surgical items after intervention is a very rare condition but, which can lead to adverse consequences for the patient in terms of morbidity and mortality, and also for the surgical team as it may trigger lawsuit. Forgotten surgical items include needles, instruments, towels and sponges, the later being the most frequent with a proportion varying from 45 to 80%. Textiloma (from Greek *textile* and *ome* = tumour) also called gossypiboma (from Latin *Gossypium* = cotton and Swahili *boma* = hiding place) is defined as the inadvertent retention of textile foreign body after surgical intervention leading to various symptoms. It is probably the oldest and most obvious error in surgery [1]. Since the first case was reported in 1884 by Wilson, hundreds of cases have been reported. According to those publications its incidence varies from 1/833 to 1/32.672 [1, 2]. The true incidence of this medical mistake is certainly higher since fear of litigation prevents many practitioners from reporting. We report the case of an abdominal textiloma in a 42 year old cameroonian woman who underwent a total abdominal hysterectomy. The textiloma was removed by another laparotomy six weeks after the causative surgery.

## Patient and observation

Mrs X is a 42-year old married teacher who consulted in our consultation for foul smelling vaginal discharge and abdominal pain., last normal menstrual period three months prior to admission.

That cramp-like and intermittent abdominal pain began four weeks prior to consultation and was associated with nausea, episodic late post-prandial vomiting and alternation between diarrhea and constipation. The patient took painkillers (paracetamol), non-steroidal anti-inflammatory drugs (ketoprofen), and antibiotics (amoxicilline-clavulanic acid and metronidazole) without real improvement. After one week of evolution the onset of foul smelling vaginal discharge motivated the use of vaginal douching with antiseptics. No episode of fever was reported. The persistence of the symptoms motivated the consultation in our hospital after the telephonic advice from her treating doctor.

The patient underwent total conservative intra-fascial abdominal hysterectomy indicated for symptomatic uterine fibroids (menometrorrhagia) six weeks prior to admission in our unit. Findings included: a big uterus (like a pregnancy of eighteen weeks) containing several leiomyomas and type B pelvic adhesions. Blood loss was estimated at 1,200 milliliters. On the sixth post-operative day she developed an intestinal sub-occlusive syndrome that was managed conservatively with nasogastric aspiration tube, antispasmodic, and painkillers. She did well and no paraclinical exam was done. The medical team assumed that etiology of the sub-occlusive syndrome was surgical adhesions. She left hospital on the tenth post-operative day with a one week prescription of antispasmodics, pain-killers and antibiotics. The first appointment with the treating doctor was set one week later but the patient didn't respect it.

In the past history, she also underwent an elective lower uterine segment cesarean section indicated for transverse lie twelve years ago and a laparotomy (salpingectomy) indicated for ruptured tubal pregnancy. Post operative courses were uneventful. She had her first menses at 14 years old and her menstrual cycle was regular at 28 days till the onset of menometrorrhagia that led to hysterectomy. She is G4P2022. Her first pregnancy was spontaneously aborted at 8 weeks without complications. The second pregnancy resulted in a normal vaginal delivery of a living girl. The third pregnancy resulted in an elective cesarean section indicated for transverse lie with a living boy. The fourth pregnancy resulted in a ruptured tubal ectopic pregnancy, managed by laparotomy and total salpingectomy. Her father died from complications of high blood pressure and diabetes. There is no other chronic medical condition nor known allergy. She does neither drink alcohol nor smoke tobacco. She has never received blood products.

Systemic enquiry revealed: foul smelling vaginal discharge, vomiting, diarrhea, constipation and abdominal distension. There were neither urinary symptoms nor fever.

On physical examination the general condition was fair and vital parameters were as follows: a blood pressure of 110/70 millimeters of mercury, a respiratory rate of 28 cycles per minute, a pulse rate of 100 pulsations per minute, a temperature of 36.9 degrees Celsius.

At the levels of the head, neck and chest, the physical findings were normal. Two scars were visible on the abdominal wall corresponding to Pfannenstiel's and median sub-umbilical incisions. Abdomen was slightly distended and there was global gardening peaking around the umbilicus. No abdominal mass was found. Mc Burney's, Rovsing's, Blumberg's and obturator's signs were absent. Bowel sounds were present. Vaginal speculum showed purulent oozing from a hole in the vaginal vault. Pelvis was painful on bimanual vaginal exam but without palpable mass.

The working diagnosis was Textiloma. Differential diagnoses were pelvic abscess, pelvic peritonitis and suppurated appendicitis with vaginal fistula.

The following work up was done: the full blood count, showed a moderate hypochromic and normomocytic anemia and normal leucocytes and platelets counts; the clotting profile (Prothrombine consumption time and kaolin-activated partial thromboplastin time) was normal. Plain abdominal radiography showed neither signs of intestinal occlusion nor pneumo-peritoneum. Abdominal ultrasound revealed an hyperechoic structure of 87 x 65 millimeters with acoustic shadow compatible with a textiloma. It also showed a homogenous hepatomegaly but no signs of peritoneal effusion.

After counseling and informed consent emergency laparotomy was carried out with the followings findings: type C adhesions between omentum and intestinal loops, and between intestinal loops and abdominal wall; textiloma made up of a big surgical sponge) eroding the neighbouring omentum and intestinal loops around the umbilicus; multiple intestinal kinkings; no intra-peritoneal collection was found.

The gestures were: median laparotomy following the previous scar, liberation of adhesions, removal of the textiloma, a partial omentectomy, a thorough verification of absence of intestinal perforation, a thorough washing of the peritoneal cavity. Two drains were left in both parieto-colic gutters and blood loss was estimated at 400 milliliters. The patient received two pints of packed red blood cells during and after surgery. She did well post-operatively and was discharged twelve days after surgery. Four months after surgery she was fine and has resumed work.

## Discussion

Risks factors of textiloma include: emergency surgery, unexpected change in surgical procedure, high body mass index, change in nursing staff during procedure, female sex, high volume of blood loss, high surgical risk, absence of meticulous surgical count of sponges instruments and needles, increased number of peri-operative personnel involved, increased number of specialty teams involved. Only the first three factors have been shown to be statistically significant by matched multivariate logistic regression. The patient in the present report underwent a planned total abdominal hysterectomy by three specialty teams: the surgeons were a senior obstetrician-gynecologist and two senior residents. The team of anesthetists was made up of two senior residents and two specialized nurses. The team of nurses included two licensed nurses. Several medical students were also present in the operation room. Blood loss was estimated at 1,200 milliliters which is enough to hide a sponge from surgeons’ sight. Non change was done in the procedure she went through and she was not obese. The surgery carried a minor risk as she was classified ASA II (American Society of Anesthesiologists). The final count of surgical items was not done at the end of the surgery and no sign out form was filled. This denotes a very common surgical malpractice or mistake in our setting. Indeed there is no clear policy for instruments, sponges and needles counts. Moreover the operating room manager who also stands as quality coordinator is not always present during procedures [3, 4].

The laps of time between causal surgery and discovery of the forgotten textile material (sponge/swab/towel/gauze) vary from few minutes to forty years. The most frequent locations of textiloma are: the abdomen (56%), the pelvis (18%) and thorax (11%) [5, 6, 7]. Retained surgical sponge consists of a cotton matrix that undergoes several changes in the body; the first day it triggers an exudative inflammatory reaction which can remain aseptic on one hand, leading to a granulation tissue after one week and to a fibrous envelop two weeks later; calcifications and encystment can occur. On the other hand, that initial exudative inflammatory site can get infected and form abscess [3, 8]. Symptoms depend on the location and the possible migration of the retained gauze and on the type of local tissue reaction (infectious or aseptic): infectious reaction leads to early manifestations and recognition of the condition whereas in cases of aseptic reaction the diagnosis can be made several decades later. Manifestations of abdominal and pelvic textiloma include fever, nausea, pain, mass, digestive fistulas, intestinal occlusion, abscess, peritonitis, foul smelling vaginal discharge [7]. Discrepancies in surgical counts can lead to early recognition of a retained sponge by radiography in the operation room in case the sponge has a radio-opaque marker [1, 2]. In the case we herein report no surgical count was reported in the post operative note.

Our patient presented early symptoms of her textiloma but the medical team failed to recognize as such. Indeed that sub-occlusive intestinal syndrome on the sixth post-operative day was caused by the forgotten gauze and should have prompted the team to carry out a plain abdominal radiography, an abdominal ultrasound or an abdominal computerized tomography. The resource-poor context and the favorable evolution under gastric drainage prevented them from requesting one of those key diagnostic investigations that should have been systematically done considering risks factors presented and the jurisprudence. After being discharge from hospital the patient did not come back for appointment with her treating doctor despite the symptoms she presented at home; instead she continuously took antibiotics and anti-inflammatory drugs for six weeks and consulted the doctor after unsuccessful auto medication for purulent vaginal discharge. She certainly carried an infected abdominal textiloma but those antibiotics prevented pelvic abscess formation and peritonitis.

On readmission, diagnosis was highly suspected after clinical examination and past history. Though the abdominal plain radiography can show indirect signs of textiloma, it is difficult to determine its topography even when a radio-opaque marker is inside. The plain abdominal radiography missed the textiloma because the forgotten gauze did not have a radio-opaque marker. On abdominal ultrasound the diagnosis of our patient was made on basis of a hyper echoic non calcified structure of 87 x 65 millimeters with acoustic shadow. This corresponds to the typical aspect of textilomas on ultrasonography. We did not request a computerized tomography scan (CT scan) of the abdomen. CT scan is very accurate in diagnosing and localizing abdominal textilomas, the typical appearance is a spongiform pattern with gas bubbles [9].

Surgical removal of the forgotten sponge is the cornerstone of textiloma care. This procedure can be done by laparotomy or by laparoscopy. Laparoscopic retrieval of textilomas is indicated in selected cases where there are no complications, where the forgotten swab is small and encapsulated [10].

Outcome after ablation of textiloma is often favorable. Morbidity is mainly due to complications. Mortality rate reported in literature is 18.9%. True mortality rate may be higher because fear of litigation prevents practitioners from reporting deaths due to this medical mistake [2].

## Conclusion

Forgetting a textile surgical item (sponge, gauze or towel) on the surgical site is a rare but serious medical mistake. Prevention can be easily achieved by adopting and implementing policies of systematic count of surgical items in operating rooms and by using only radio-opaque sponges. Textiloma is the differential diagnosis of all abdominal and pelvic masses and occlusive syndromes in patient with previous surgeries at those sites and should always be considered. Hiding the diagnosis to a patient in her family is not ethically correct but the team did not want to leave space for claim. Given that the majority of Africans live below the poverty line, we must be more careful about the count of compresses and other instruments before, during and after surgery. This avoids the costs associated with morbidity and prosecution.
